# A proposed new Japanese classification of synchronous peritoneal metastases from colorectal cancer: A multi‐institutional, prospective, observational study conducted by the Japanese Society for Cancer of the Colon and Rectum

**DOI:** 10.1002/ags3.12679

**Published:** 2023-04-23

**Authors:** Hirotoshi Kobayashi, Kenjiro Kotake, Masayasu Kawasaki, Yukihide Kanemitsu, Yusuke Kinugasa, Hideki Ueno, Kotaro Maeda, Takeshi Suto, Michio Itabashi, Kimihiko Funahashi, Heita Ozawa, Fumikazu Koyama, Shingo Noura, Hideyuki Ishida, Masayuki Ohue, Tomomichi Kiyomatsu, Soichiro Ishihara, Keiji Koda, Hideo Baba, Kenji Kawada, Yojiro Hashiguchi, Takanori Goi, Yuji Toiyama, Naohiro Tomita, Eiji Sunami, Yoshito Akagi, Jun Watanabe, Kenichi Hakamada, Goro Nakayama, Kenichi Sugihara, Yoichi Ajioka

**Affiliations:** ^1^ Department of Surgery Tokyo Metropolitan Hiroo Hospital Tokyo Japan; ^2^ Department of Surgery Teikyo University Hospital Mizonokuchi Kanagawa Japan; ^3^ Department of Surgery Sano City Hospital Tochigi Japan; ^4^ Department of Surgery Bell Land General Hospital Sakai Japan; ^5^ Department of Colorectal Surgery National Cancer Center Hospital Tokyo Japan; ^6^ Department of Gastrointestinal Surgery Tokyo Medical and Dental University Tokyo Japan; ^7^ Department of Surgery National Defense Medical College Tokorozawa Japan; ^8^ International Medical Center Fujita Health University Hospital Toyoake Japan; ^9^ Department of Gastroenterological Surgery Yamagata Prefectural Central Hospital Yamagata Japan; ^10^ Department of Surgery Institute of Gastroenterology, Tokyo Women's Medical University Tokyo Japan; ^11^ Department of General and Gastroenterological Surgery Toho University Omori Medical Center Tokyo Japan; ^12^ Department of Surgery Tochigi Cancer Center Utsunomiya Japan; ^13^ Department of Surgery Nara Medical University Kashihara Japan; ^14^ Department of Surgery Osaka Rosai Hospital Sakai Japan; ^15^ Department of Digestive Tract and General Surgery Saitama Medical Center, Saitama Medical University Kawagoe Japan; ^16^ Department of Gastroenterological Surgery Osaka International Cancer Institute Osaka Japan; ^17^ Department of Colorectal Surgery National Center for Global Health and Medicine Tokyo Japan; ^18^ Department of Surgical Oncology The University of Tokyo Hospital Tokyo Japan; ^19^ Department of Surgery Teikyo University Chiba Medical Center Ichihara City Chiba Japan; ^20^ Department of Gastroenterological Surgery Graduate School of Medical Sciences, Kumamoto University Kumamoto Japan; ^21^ Department of Gastrointestinal Surgery Graduate School of Medicine, Kyoto University Kyoto Japan; ^22^ Department of Surgery Teikyo University School of Medicine Tokyo Japan; ^23^ First Department of Surgery University of Fukui Fukui Japan; ^24^ Division of Reparative Medicine, Department of Gastrointestinal and Pediatric Surgery, Institute of Life Sciences Mie University Graduate School of Medicine Tsu Mie Japan; ^25^ Division of Lower Gastrointestinal Surgery, Department of Gastroenterological Surgery Hyogo Medical University Nishinomiya Hyogo Japan; ^26^ Department of Surgery Kyorin University School of Medicine Tokyo Japan; ^27^ Department of Surgery Kurume University School of Medicine Kurume Japan; ^28^ Department of Surgery Gastroenterological Center, Yokohama City University Medical Center Yokohama Japan; ^29^ Department of Gastroenterological Surgery Hirosaki University Graduate School of Medicine Aomori Japan; ^30^ Department of Gastroenterological Surgery (Surgery II) Nagoya University Graduate School of Medicine Nagoya Japan; ^31^ Tokyo Medical and Dental University Tokyo Japan; ^32^ Division of Molecular and Diagnostic Pathology, Graduate School of Medical and Dental Sciences Niigata University Niigata Japan

**Keywords:** classification, colorectal cancer, JSCCR, peritoneal metastasis, R0 resection

## Abstract

**Aim:**

To establish a new Japanese classification of synchronous peritoneal metastases from colorectal cancer.

**Methods:**

This multi‐institutional, prospective, observational study enrolled patients who underwent surgery for colorectal cancer with synchronous peritoneal metastases. Overall survival rates were compared according to the various models using objective indicators. Each model was evaluated by Akaike's information criterion (AIC). The region of peritoneal metastases was evaluated by the peritoneal cancer index (PCI).

**Results:**

Between October 2012 and December 2016, 150 patients were enrolled. The AIC of the present Japanese classification was 1020.7. P1 metastasis was defined as confined to two regions. The minimum AIC was obtained with the cutoff number of 10 or less for P2 metastasis and 11 or more for P3 metastasis. As for size, the best discrimination ability between P2 and P3 metastasis was obtained with a cutoff value of 3 cm. The AIC of the proposed classification was 1014.7. The classification was as follows: P0, no peritoneal metastases; P1, metastases localized to adjacent peritoneum (within two regions of PCI); P2, metastases to distant peritoneum, number ≤10 and size ≤3 cm; P3, metastases to distant peritoneum, number ≥11 or size >3 cm; P3a, metastases to distant peritoneum, number ≥11 and size ≤3 cm, or number ≤10 and size >3 cm; P3b, metastases to distant peritoneum, number ≥11 and size >3 cm.

**Conclusion:**

This objective classification could improve the ability to discriminate prognosis in patients with synchronous peritoneal metastases from colorectal cancer.

## Graphical abstract

The present study demonstrated a new objective classification for peritoneal metastasis from colorectal cancer.

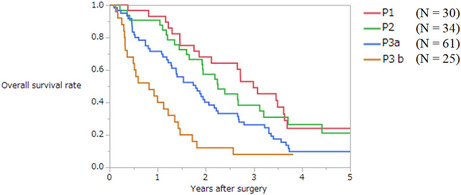

## 1 INTRODUCTION

Colorectal cancer is the second most common cause of cancer death in the United States and Japan.^1^, ^2^ Furthermore, the incidence of colorectal cancer has been increasing in Japan.^3^, ^4^

Peritoneal metastasis is one of the factors associated with a poor prognosis in patients with colorectal cancer and is found in approximately 5% of primary colorectal cancer cases.^5^ Colorectal cancer with synchronous peritoneal metastases is classified as Stage IVC in the current AJCC Cancer Staging Manual.^6^

The Japanese classification of colorectal carcinoma is published by the Japanese Society for Cancer of the Colon and Rectum (JSCCR).^7^ In the present Japanese classification, synchronous peritoneal metastasis was classified as follows:

P0, no peritoneal metastasis;
P1, metastasis localized to adjacent peritoneum;
P2, limited metastasis to distant peritoneum; and.
P3, diffuse metastasis to distant peritoneum.

Although this classification is easy to use, it seems less objective.

On the other hand, in specialized centers for peritoneal diseases, the peritoneal cancer index (PCI) has been used worldwide.^8^ The PCI classifies the abdominal cavity into 13 regions. In each region, 0 to 3 points are given according to the size of peritoneal metastases. Therefore, the maximum PCI score is 39. The PCI has superior objectivity, but it seems cumbersome for general surgeons.

The aim of this study was to establish a new objective classification of peritoneal metastases from colorectal cancer in a multi‐institutional, prospective, observational study.

## 2 METHODS

### 2.1 Study design

The 28 member hospitals of the JSCCR were involved in this multi‐institutional, prospective, observational study. These hospitals joined a committee of the JSCCR, named “Grading of peritoneal metastasis from colorectal cancer.” Patients who underwent surgery for colorectal cancer with synchronous peritoneal metastases between October 2012 and December 2016 were enrolled. Clinical and pathological information was registered within 3 months after surgery. Prognostic information was collected 3 years after surgery. Written, informed consent was obtained from all patients before enrollment. Since synchronous peritoneal metastases from colorectal cancer are often found accidentally during surgery, written informed consent could be obtained after surgery in such cases. The ethics committees of the Japanese Society of the Colon and Rectum and each institution approved this study. In this study, tumor location was classified into right colon (velmiformis, cecum, ascending colon, transverse colon), left colon (descending colon, sigmoid colon), and rectum (RS, Ra, Rb).

### 2.2 Surgical procedure

The surgical procedure was not determined by the protocol of this study, because there are a variety of conditions in patients with synchronous peritoneal metastases. Each surgeon made a decision about primary tumor resection and peritoneal metastasis resection.

### 2.3 Data collection

All data were collected prospectively. The preoperative data included physical information, blood tests, and preoperative diagnosis. The information regarding peritoneal metastases included both the Japanese classification and the PCI. The size and number of peritoneal metastases in each region were recorded. Surgical procedures and other pathological information were sent within 3 months after surgery. Information regarding postoperative chemotherapy and outcomes was collected at least 3 years after surgery.

### 2.4 Statistical analysis

Various classification models were constructed by combinations of region, number, and size of peritoneal metastases and compared. The discrimination ability of each model was evaluated by Akaike's information criterion. Actuarial survival after surgery was depicted by Kaplan–Meier curves. The log‐rank test was used to compare overall survivals. Differences in continuous variables were compared using the Kruskal–Wallis test.

JMP 13 software (SAS Institute Japan, Ltd.) was used for data analysis. The data are expressed as medians and range or numbers of patients and percentages (%). A *p* value less than 0.05 was taken to indicate significance in this study.

## 3 RESULTS

### 3.1 Patients' characteristics

Table 1 shows the patients' characteristics for the entire cohort. A total of 150 patients were enrolled in this study; their median age was 66 years. The location of the primary tumor was the right colon in approximately half of the patients. The primary tumor was not resected in 24 patients in whom pathological T and N factors were not available. Cytoreductive surgery with hyperthermic intraperitoneal chemotherapy (HIPEC) was performed in only two patients.

**TABLE 1:** Patients' characteristics.

	Entire cohort (*N* = 150) (%)
Age	66 (30–89)
Gender	
Male	84 (56)
Female	66 (44)
Location of primary tumor	
Right colon	79 (53)
Left colon	44 (29)
Rectum	27 (18)
Histologic type of primary tumor	
Well or mod	110 (73)
Others	36 (24)
Unknown	4 (3)
Primary tumor resection	
Absent	24 (16)
Present	126 (84)
T‐category	
T3	12 (8)
T4a	86 (57)
T4b	28 (19)
Unknown	24 (16)
N‐category	
N0	29 (19)
N1a	18 (12)
N1b	24 (16)
N2a	27 (18)
N2b	28 (19)
Unknown	24 (16)
Peritoneal metastasis	
P1	30 (20)
P2	57 (38)
P3	63 (42)
PCI	4 (1–29)
Distant metastasis	
Absent	62 (41)
Present	88 (59)
Residual tumor	
R0	32 (21)
R1	5 (3)
R2	113 (75)

Well or mod: well or moderately differentiated adenocarcinoma.

Right colon: velmiformis, cecum, ascending colon, transverse colon.

Left colon: descending colon, sigmoid colon.

Age, PCI: median (range).

Abbreviation: PCI, peritoneal cancer index.

### 3.2 Peritoneal metastases

According to the present Japanese classification, the number of patients with synchronous peritoneal metastases was 30 (20%) in P1, 57 (38%) in P2, and 63 (42%) in P3. The median PCI was 4 (1–29).

### 3.3 Survival

**FIGURE 1:**
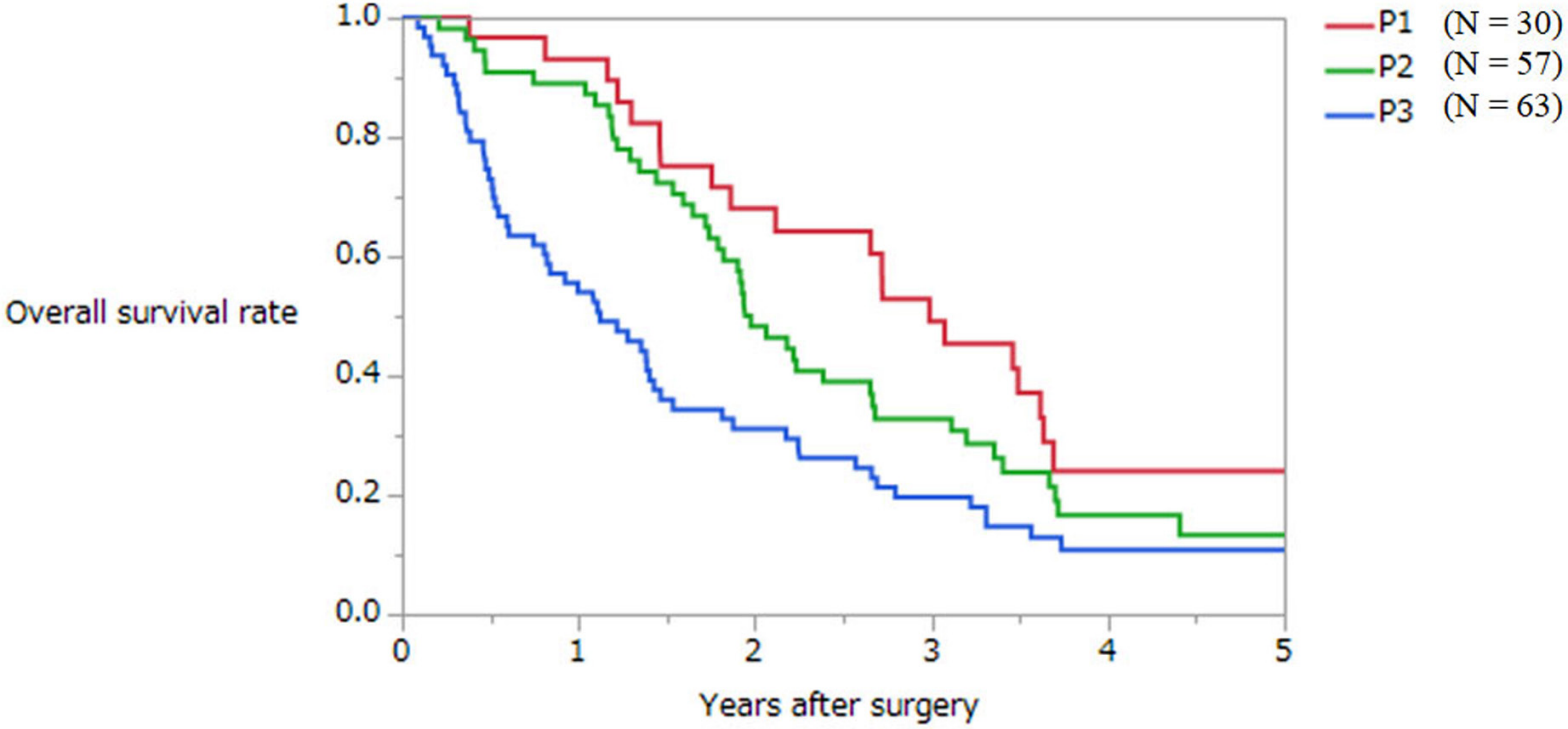
Overall survival curves of patients with synchronous peritoneal metastases from colorectal cancer according to the present Japanese classification.

The median survival time (MST) of the entire cohort was 1.9 (0.1–6.5) years. The MST of patients with P1, P2, and P3 metastases was 3.0 years, 2.0 years, and 1.1 years, respectively (*p* = 0.0013, Figure 1). The 3‐year survival rate of patients with P1, P2, and P3 metastases was 49%, 33%, and 20%, respectively. The AIC of the present Japanese classification was 1020.7.

### 3.4 Clinical question 1 (range of P1 metastases)

One of the clinical questions was the range of P1 metastases, i.e., localized peritoneal metastases. In this study, 30 patients were classified as having P1 metastases. Of the 30 P1 metastases, 23 (77%) were confined to one area, and seven (23%) were confined to two areas. Therefore, all P1 metastases were confined to one or two areas.

### 3.5 Clinical question 2 (cutoff number between P2 and P3 metastases)

**FIGURE 2:**
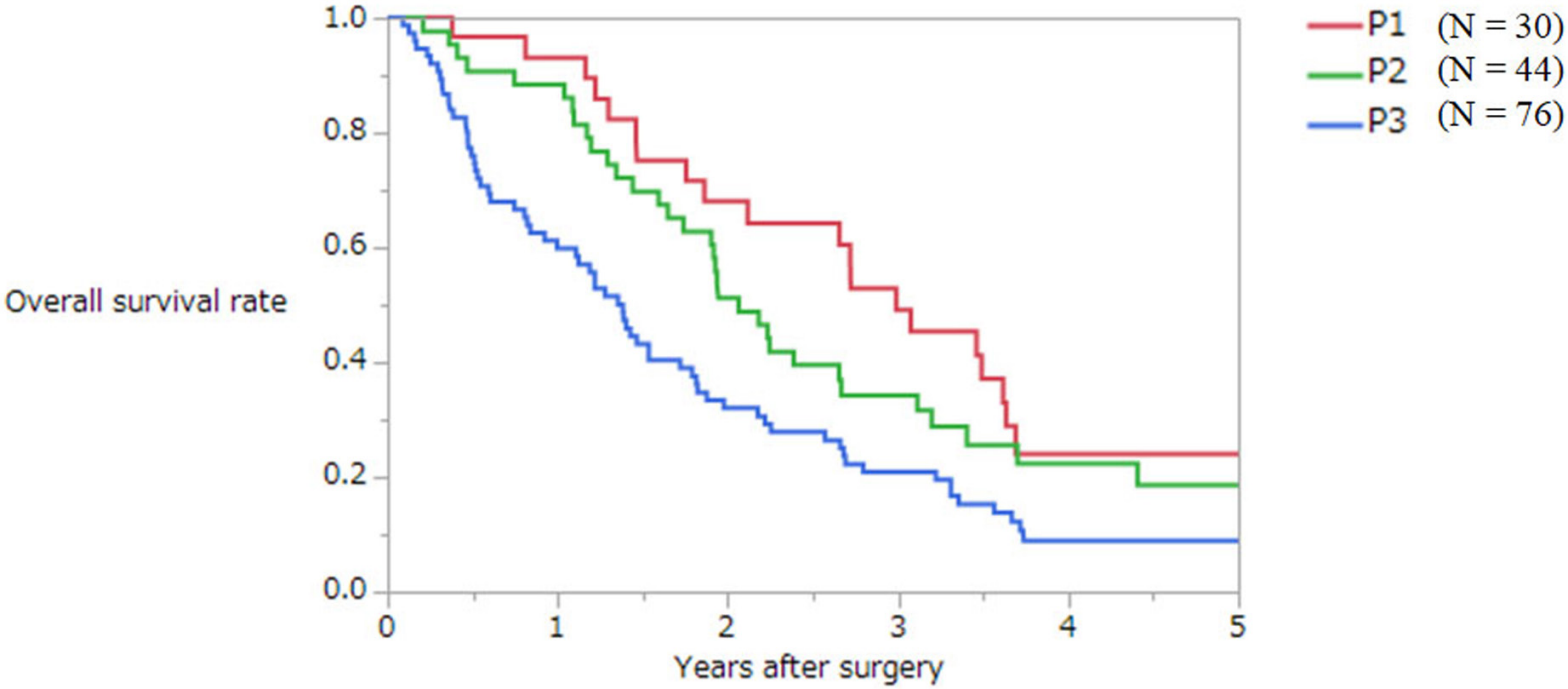
Overall survival curves of patients with synchronous peritoneal metastases from colorectal cancer when the number of P2 peritoneal metastases is defined as 10 or less.

Another clinical question was the cutoff number between P2 and P3 metastases. The AIC was evaluated according to the number of peritoneal metastases. The AIC according to the number of P2 ≤9, ≤10, ≤19, and ≤20 was 1023.3, 1020.7, 1021.2, and 1022.2, respectively. Therefore, the best cutoff number of P2 metastases was 10. Figure 2 shows the survival curves when P2 was defined as metastases to distant peritoneum (number of peritoneal metastases ≤10, Figure 2).

### 3.6 Clinical question 3 (size of peritoneal metastases)

**FIGURE 3:**
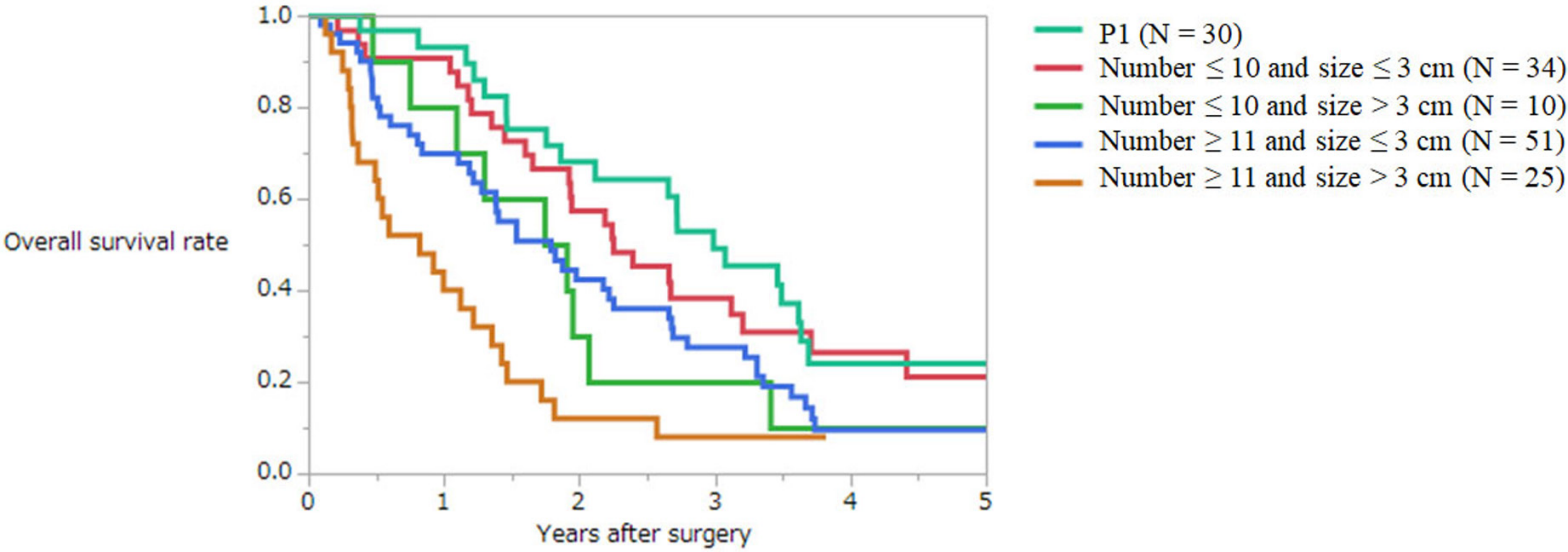
Overall survival curves of patients with synchronous peritoneal metastases from colorectal cancer when P2 and P3 are classified by both the number and size of the peritoneal metastases.

**FIGURE 4:**
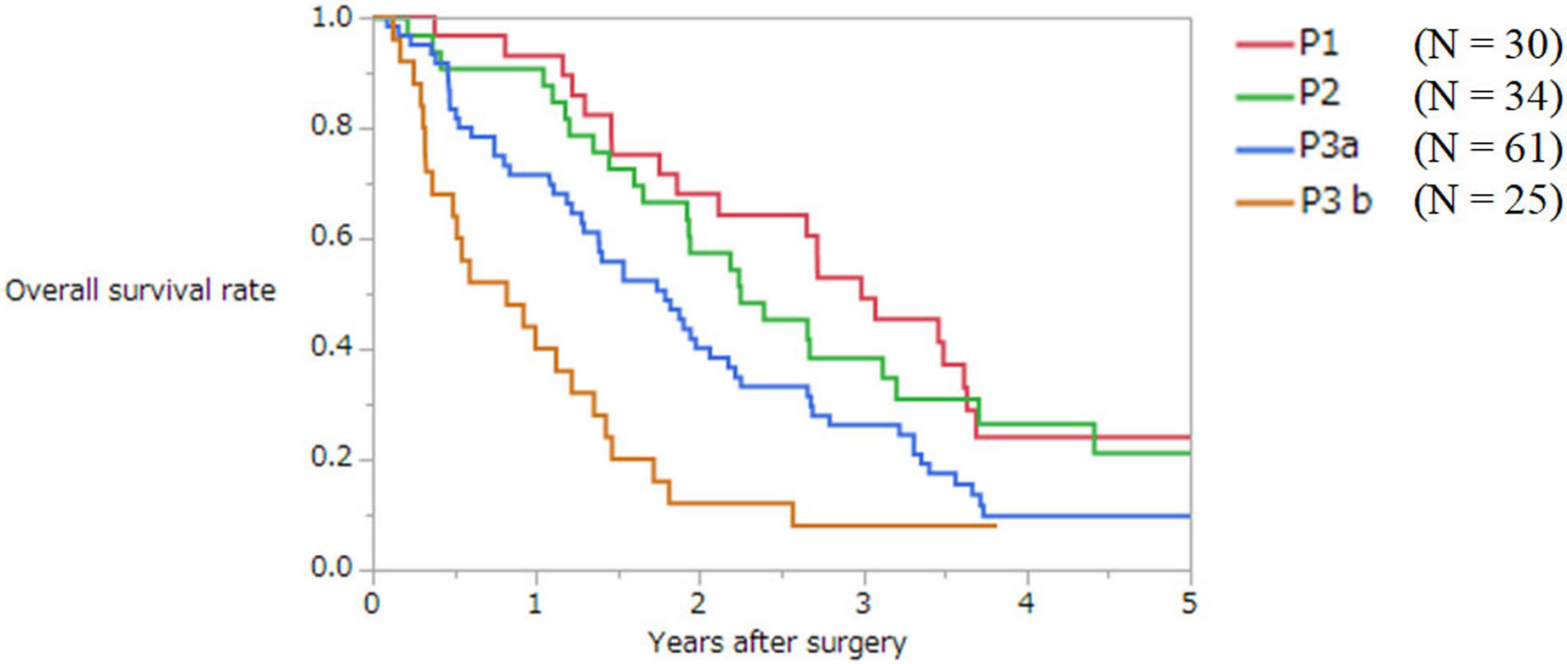
Overall survival curves of patients with synchronous peritoneal metastases from colorectal cancer according to the proposed classification.

The present Japanese classification does not include the concept of size of peritoneal metastases. As a first step, the AIC was investigated according to the size of peritoneal metastases. The AICs of cutoff values of 5, 10, 20, 30, and 50 mm were 1031.4, 1030.2, 1021.3, 1021.3, and 1029.8, respectively. The *p* value of the log‐rank test according to each size was 0.59, 0.23, 0.0011, 0.0005, and 0.15, respectively. A new classification model was then created in which the concepts of both number and size (cutoff values of 20 and 30 mm) of peritoneal metastases were added to P2 and P3 metastases. Figure 3 shows the survival curves according to the combination of size (cutoff value 30 mm) and number (cutoff value 10). The survival curve of patients with peritoneal metastases of 10 or less and larger than 3 cm was similar to that with peritoneal metastases of 11 or more and 3 cm or smaller in size. Therefore, these two groups were subclassified as one group. The AIC of this model was 1014.7. The same model was created using the cutoff value of 20 mm. Its AIC was 1015.0. Therefore, the model with the best ability to discriminate prognosis in patients with synchronous peritoneal metastases was as follows (Figure 4):

P0, no peritoneal metastasis;
P1, metastasis localized to adjacent peritoneum (within two regions of PCI);
P2, metastasis to distant peritoneum, number ≤10 and size ≤3 cm;
P3, metastasis to distant peritoneum, number ≥11 or size >3 cm;
P3a, metastasis to distant peritoneum, number ≥11 and size ≤3 cm, or number ≤10 and size >3 cm; and.
P3b, metastasis to distant peritoneum, number ≥11 and size >3 cm.

The median PCI score of the proposed P1, P2, P3a, and P3b was 2 (1–5), 3 (1–9), 9 (2–26), and 19 (4–29), respectively (*p* < 0.0001).

The proposed classification reflected the prognosis of patients with and without distant metastasis (*p* = 0.027 and 0.0017).

### 3.7 R0 resection

The R0 resection rate of P1, P2, and P3 metastases according to the present Japanese classification was 53%, 26%, and 1.6%, respectively (*p* < 0.0001). On the other hand, the R0 resection rate of P1, P2, and P3 metastases of the above‐proposed classification was 53%, 32%, and 5.8%, respectively (*p* < 0.0001).

### 3.8 Chemotherapy

Thirty out of 38 patients with curative resection received adjuvant chemotherapy. Among them, 22 patients received oxaliplatin‐based chemotherapy. As for the 112 patients with non‐curative resection, 94 patients (80%) received chemotherapy. The regimens were as follows: FOLFOXIRI (*n* = 1), oxaliplatin‐based (*n* = 61), irinotecan‐based (*n* = 17), and monotherapy (*n* = 15). Molecular‐targeted agent was also used in most patients.

## 4 DISCUSSION

It has been reported that the present Japanese classification of peritoneal metastases from colorectal cancer is easy to use and useful in predicting prognosis.^5^, ^9^, ^10^, ^11^, ^12^ In addition, the present Japanese classification is also useful in determining whether synchronous peritoneal metastases should be resected during surgery. The present Japanese classification predicted the prognosis of patients with synchronous peritoneal metastases from colorectal cancer very well. On the other hand, it has been pointed out that the present Japanese classification seems less objective. Therefore, a major aim of this study was to add objectivity to the present Japanese classification. At the same time, whether the concept of the size of peritoneal metastases could improve the ability to discriminate prognosis was also validated. To ensure consistency with the world standard, the region of peritoneal metastases was determined according to the PCI in the present study.

The first clinical question related to the difference in extent between P1 and P2 metastases in the present Japanese classification. P1 metastases are limited to adjacent peritoneum. In contrast, P2 metastases are metastases limited to distant peritoneum. It has been difficult to explain the difference in extent between P1 and P2 metastases objectively. The present study demonstrated that P1 metastases were confined to one or two regions. In other words, the Japanese surgeons judged P1 metastases as peritoneal metastases confined to one or two adjacent regions. Therefore, it is reasonable to define P1 as peritoneal metastases localized to adjacent peritoneum within two regions of the PCI.

The second clinical question related to the cutoff number of peritoneal metastases between P2 and P3 metastases. The present Japanese classification defines P2 and P3 metastases as limited and diffuse metastases to distant peritoneum, respectively. The present study demonstrated that the AIC was at a minimum when the cutoff number of P2 and P3 metastases was 10. Kawasaki et al.^13^ reported that when the number of peritoneal metastases was 19 or less, many surgeons classified them as P2 metastases. Kawasaki's classification was validated from the viewpoint of prognosis. However, there was no difference in survival between P1 and P2 metastases using Kawasaki's classification (data not shown). Meanwhile, Kobayashi et al.^9^ reported that it was appropriate that P3 metastases be defined as >10 peritoneal metastases in their multi‐institutional, retrospective study. Their cutoff number between P2 and P3 metastases was consistent with that in the present study.

The third clinical question related to whether the concept of size of peritoneal metastases could improve the ability of the Japanese classification to discriminate prognosis. The PCI uses the cutoff sizes of 5 and 50 mm. However, in the current study, there was no difference in survival according to the sizes of 5 and 50 mm. The subclassification using the cutoff size of 30 mm most effectively improved the ability to discriminate prognosis in the current study.

The present Japanese classification has been used as an indicator for R0 resection in patients with synchronous peritoneal metastases. In the Japanese guidelines for the treatment of colorectal cancer, complete resection is strongly recommended for P1 metastases.^14^ Complete resection is recommended for P2 metastases when they are easily resectable. The R0 resection rate for P1 and P2 metastases using the proposed classification in the current study was comparable to that in the present Japanese classification.

There are some limitations in this study. First, although the data were collected prospectively using the same case report form, the surgical procedure was dependent on each surgeon. Most synchronous metastases were found accidentally during surgery. Since surgeons determined the surgical procedure during surgery, the treatment strategy may have differed by surgeon and institution.

Second, chemotherapy after surgery for patients with synchronous peritoneal metastases from colorectal cancer might vary from institution to institution. Since this was an observational study, treatment strategy including surgical procedure and chemotherapy depended on each attending physician. Current chemotherapy was performed if tolerable. However, various regimens were used in this study.

Third, few patients underwent cytoreductive surgery with HIPEC. Therefore, the current proposed classification would be helpful in most Japanese institutions where such aggressive treatment is not performed. Cytoreductive surgery with HIPEC is performed at specialized centers in the world. However, the few patients with cytoreductive surgery with HIPEC in the current study proved that such treatment was not common in Japan. Although the effectiveness of HIPEC was reported in some studies,^15^, ^16^ it remains controversial. A multicenter, randomized, open‐label, phase 3 trial, PRODIGE 7, failed to demonstrate the superiority of cytoreductive surgery with HIPEC compared to cytoreductive surgery alone.^17^ At present, cytoreductive surgery with HIPEC should be performed in specialized centers. Meanwhile, since cytoreductive surgery with HIPEC is not common in most Japanese hospitals, the proposed classification would be useful in determining the surgical procedure and predicting the prognosis.

Fourth, the concept of the size of peritoneal metastases was added to the proposed classification. Although the ability to discriminate prognosis was improved, the proposed classification seemed more complicated. Whether the proposed classification including the size of peritoneal metastases is actually acceptable and easy to use for general surgeons should be carefully validated.

Fifth, the proposed classification was established using the data of synchronous peritoneal metastases from colorectal cancer. Therefore, whether this proposed classification is appropriate for metachronous peritoneal metastases from colorectal cancer should be validated in the future.

Sixth, the number of patients in this study was relatively small. Since synchronous peritoneal metastasis is relatively rare, it was difficult to collect a large number of patients prospectively under the standardized manner. The proposed classification should be validated in the future.

In conclusion, this study succeeded in establishing a new objective classification of synchronous peritoneal metastases from colorectal cancer while following the present Japanese classification. In addition, the concept of the size of peritoneal metastases improved the ability to discriminate prognosis.

## ETHICS STATEMENT

Approval of the research protocol: The study protocol followed the ethical guidelines of the 2008 Declaration of Seoul and was approved by the Institutional Review Board at the JSCCR and each institution.

Informed Consent: A written informed consent was obtained from each patient.

Registry and the Registration No. of the study/Trial: N/A.

Animal Studies: N/A.

## CONFLICT OF INTEREST STATEMENT

Hideki Ueno, Hideo Baba, Jun Watanabe, and Kenichi Hakamada are editorial board members of *Annals of Gastroenterological Surgery*.
